# Magnetic resonance spectroscopy and the menstrual cycle: A multi-centre assessment of menstrual cycle effects on GABA & GSH

**DOI:** 10.1016/j.jneumeth.2025.110430

**Published:** 2025-03-19

**Authors:** Yulu Song, James J. Prisciandaro, Dace Apšvalka, Mae Bernard, Adam Berrington, Miguel Castelo-Branco, Mark K. Britton, Marta M. Correia, Koen Cuypers, Aleksandra Domagalik, Ulrike Dydak, Niall W. Duncan, Gerard E. Dwyer, Tao Gong, Ian Greenhouse, Katarzyna Hat, Melina Hehl, Shiori Honda, Chris Horton, Steve C.N. Hui, Stephen R. Jackson, Daniella L. Jones, Maren S. Klan, In Kyoon Lyoo, Marius O. Mada, Bronte V. McNamara, Paul G. Mullins, Emlyn Muska, Shinichiro Nakajima, Hayami Nishio, Andreia C. Pereira, Eric C. Porges, Michelle Rowsell, Rubi Ruopp, Destin D. Shortell, Caitlin M. Smith, Stephan Swinnen, Antonia Šušnjar, Lin-Yuan Tseng, Ines R. Violante, Sujung Yoon, Richard A.E. Edden, Katherine Dyke

**Affiliations:** a Russell H. Morgan Department of Radiology and Radiological Science, The Johns Hopkins University School of Medicine, Baltimore, MD, USA; b F. M. Kirby Research Center for Functional Brain Imaging, Kennedy Krieger Institute, Baltimore, MD, USA; c Addiction Sciences Division, Department of Psychiatry and Behavioral Sciences, Medical University of South Carolina, Charleston, USA; d MRC Cognition and Brain Sciences Unit, University of Cambridge, UK; e School of Psychology and Sports Sciences, Bangor University, Bangor, UK; f Sir Peter Mansfield Imaging Centre, School of Physics and Astronomy, University of Nottingham, Nottingham, UK; g Institute for Nuclear Sciences Applied to Health (ICNAS), Coimbra Institute for Biomedical Imaging and Translational Research (CIBIT), University of Coimbra, Portugal; h Center for Cognitive Aging and Memory and McKnight Brain Institute, University of Florida, Gainesville, FL, USA; i Neuroplasticity and Movement Control Research Group, Rehabilitation Research Institute (REVAL), Hasselt University, Diepenbeek, Belgium; j Movement Control & Neuroplasticity Research Group, Department of Movement Sciences, Group Biomedical Sciences, KU Leuven, Heverlee, Belgium; k Leuven Brain Institute (LBI), KU Leuven, Leuven, Belgium; l Centre for Brain Research, Jagiellonian University, Krakow, Poland; m School of Health Sciences, Purdue University, West Lafayette, IN, USA; n Department of Radiology and Imaging Sciences, Indiana University School of Medicine, Indianapolis, IN, USA; o Graduate Institute of Mind, Brain and Consciousness, Taipei Medical University, Taipei, Taiwan; p Brain and Consciousness Research Centre, TMU Shuang-Ho Hospital, New Taipei City, Taiwan; q Department of Biological and Medical Psychology, University of Bergen, Bergen, Norway; r NORMENT Centre of Excellence, Haukeland University Hospital, Bergen, Norway; s Departments of Radiology, Shandong Provincial Hospital Affiliated to Shandong First Medical University, Jinan, Shandong, China; t Departments of Radiology, Shandong Provincial Hospital, Cheeloo College of Medicine, Jinan, Shandong, China; u Institute of Neuroscience, University of Oregon, Eugene, OR 97403, USA; v Department of Human Physiology, University of Oregon, Eugene, OR 97403, USA; w Doctoral School in the Social Sciences, Jagiellonian University, Poland; x Consciousness Lab, Institute of Psychology, Jagiellonian University, Kraków, Poland; y Translational MRI, Department of Imaging & Pathology, KU Leuven, Leuven, Belgium; z Department of Neuropsychiatry, Keio University, School of Medicine, Tokyo, Japan; aa Developing Brain Institute, Children’s National Hospital, Washington, DC, USA; ab Department of Radiology, The George Washington University School of Medicine and Health Sciences, Washington, DC, USA; ac Department of Pediatrics, The George Washington University School of Medicine and Health Sciences, Washington, DC, USA; ad School of psychology, University of Nottingham, Nottingham, UK; ae Neurotherapeutics Ltd, The Ingenuity Centre, University of Nottingham Innovation Park, Triumph Road, Nottingham NG7 2TU, UK; af Institute of Mental Health, School of Medicine, University of Nottingham, Nottingham, UK; ag School of Psychology, University of Surrey, Guildford GU2 7XH, UK; ah Ewha Brain Institute, Graduate School of Pharmaceutical Sciences, and Dept. of Brain and Cognitive Sciences, Ewha Womans University, Seoul, South Korea; ai Department of Clinical and Health Psychology, College of Public Health and Health Professions, University of Florida, 1225 Center Drive, Gainesville, FL 32611, USA; aj Multimodal Imaging Group, Centre for Addiction and Mental Health, University of Toronto, Canada; ak Weldon School of Biomedical Engineering, Purdue University, West Lafayette, IN 4796, USA; al School of Biomedical Engineering and Imaging Sciences, King’s College London, London, UK

**Keywords:** Gamma-aminobutyric acid (GABA), Glutathione (GSH), Magnetic resonance spectroscopy (MRS), Hadamard Encoding and Reconstruction of MEGA-Edited Spectroscopy (HERMES), Menstrual cycle, Oestrogen

## Abstract

**Background:**

Gamma-aminobutyric acid (GABA) and glutathione (GSH) play a significant role in the functioning of a healthy brain and can both be quantified using magnetic resonance spectroscopy (MRS). Several small-scale studies have suggested MRS measured GABA may fluctuate with the menstrual cycle, but the effects on GSH are unknown. Utilising recent developments in MRS acquisition, this multi-lab study explores this issue across 4 distinctive brain regions.

**New methods:**

Data were analysed from 12 independent sites from which a total of 30 women were scanned during three phases of their menstrual cycle corresponding to early follicular, ovulation and mid luteal phases. HERMES and HERCULES sequences were used to measure GABA and GSH in voxels located in the left motor cortex, left posterior insular, medial parietal and medial frontal. Linear mixed models were used to assess the variability contributed by site, participant and menstrual cycle phase.

**Results:**

Similar variance was attributed to site and menstrual cycle phase for both GABA and GSH data. No systematic changes in GABA or GSH were revealed for any voxel as a consequence of menstrual cycle phase.

**Comparison with existing methods:**

Despite our larger sample size and inclusion of more brain regions we fail to replicate previous findings of GABA change as a consequence of menstrual cycle phase. We also show for the first time that MRS measures of GSH so not significantly alter with cycle.

**Conclusions:**

Our findings suggest that the menstrual cycle has minimal impact on MRS measures of GABA and GSH. The presence of a menstrual cycle should not be used as justification for exclusion of women in MRS studies.

## 1. Introduction

Gamma-aminobutyric acid (GABA) is the primary inhibitory neurotransmitter in the human brain and plays a critical role in key functions including learning, memory, motor coordination and sensory processing (Koh et al., 2023). Alterations in GABA signalling have been implicated in numerous clinical conditions, making the reliable and accurate measurement of GABA of great interest for studying the brain in health and disease. Another neuro-metabolite of interest to many working within clinical fields is glutathione (GSH). GSH has numerous fundamental functions in the central nervous system, including redox maintenance, and impairments in GSH function have been linked to ageing and neurological diseases such as stroke and Alzheimer’s disease (Iskusnykh et al., 2022). Levels of both GABA and GSH can be quantified non-invasively using proton magnetic resonance spectroscopy (MRS), and several studies have been conducted to establish the reliability of these measures.

The stability of MRS measures of GABA has typically been found to be moderate-to-good within healthy subjects; however, the degree to which this is true depends on the sequences used, the region studied and the temporal window in which data is acquired. Measures acquired from sensorimotor regions using the MEGA-PRESS sequence (Mescher et al., 1998) have been shown to correlate well across a three-month interval (Ferland et al., 2019). In contrast, measures from the occipital cortex taken 1–7 days apart using MEGA-PRESS (Mescher et al., 1998), JPRESS (Ryner et al., 1995) and PRESS (Bottomley, 1987) sequences showed variable levels of test-retest reliability, with MEGA-PRESS coming out best (Baeshen et al., 2020). More recently, the Hadamard Encoding and Reconstruction of MEGA-Edited Spectroscopy (HERMES) sequence, which enables simultaneous acquisition of GABA and GSH, was shown to have similar reliability to MEGA-PRESS sequences (Prisciandaro et al., 2020). Overall, MRS measures have been shown to have moderate-good test-retest reliability. These studies have been conducted typically within a restrictive age range, in mixed samples of men and women, often not sex-disaggregated.

Interestingly, it has been suggested that average levels of MRS measured GABA may differ between biological females and males. One study with 5 female/7 male participants reported higher GABA levels within the dorsolateral prefrontal cortex of male participants (O’Gorman et al., 2011). Another (7 female/7 male participants) reported higher levels of GABA within the anterior cingulate cortex of male compared to females (Sheffield and Noseworthy, 2010), and Sanacora et al. (1999) found depressed (N = 6) and non-depressed (N = 7) women had higher levels of GABA within the occipital cortex than their male counterparts. Why these differences occur, and whether they are simply artefact of small-n studies, has not been sufficiently explored, but it is possible that differing hormonal levels could contribute.

Cyclic changes in hormone levels have historically been, and in some instances, continue to be cited as reasonable premises for conducting research on male-only samples. However, there are numerous limitations and ethical issues with this approach (Beery and Zucker, 2011), and surprisingly few systematic explorations. Far from being unpredictable, changes in hormone levels across the menstrual cycle are well-characterised. Broadly speaking, the menstrual cycle can be conceptualised as two distinct phases: follicular and luteal. The follicular phase starts with the onset of menstruation and ends with ovulation, at which point the luteal phase starts. The average cycle length is approximately 29 days, although significant variability in cycle length is common (Fehring et al., 2006). For example, recent work highlights links between average cycle variability and length with age (Bull et al., 2019). Different phases of the menstrual cycle are characterised by distinct rises and falls in hormonal levels. The early follicular phase is characterised by low levels of oestrogen and progesterone (Windham et al., 2002), with oestrogen rising during the late follicular phase and peaking just before ovulation (Backstrom et al., 1982). The peak in oestrogen levels coincides with a sharp, subsequent increase in luteinizing hormone (LH) which peaks during ovulation (Backstrom et al., 1982). The luteal phase, which follows and typically lasts approximately 14 days (Reed and Carr, 2000) is characterised by a gradual increase in progesterone levels, which peaks on average 6–8 days following ovulation. Following this peak, oestrogen initially rapidly decreases alongside LH, then gradually rises to a moderate level before falling (simultaneously with a decrease in progesterone) ahead of the start of a new follicular phase.

Several studies have sought to explore how the menstrual cycle may impact MRS measures of GABA. Some have found a significant increase in GABA around ovulation (De Bondt et al., 2015), whereas others have reported higher levels during the follicular phase compared to luteal in healthy women (Epperson et al., 2002; Harada et al., 2011). These patterns have been reported to be altered in women with premenstrual dysphoric disorder and smokers (Epperson et al., 2002, 2005), and have not been found in all studies (Chrzan et al., 2013). At present, the evidence base relies on a modest number of studies (See Table 1 for a more detailed summary) with relatively small sample sizes, often acquiring data from only one brain region and with a low number of spectral transients acquired (resulting in low SNR).

To address the current debate in GABA-related fluctuations during the menstrual cycle and the possibility of region-specific profiles confounding previous findings a larger study is needed. Thus, here we report the findings of a multi-centre study specifically designed to investigate menstrual cycle-related changes in GABA levels. In addition, as changes in plasma levels of GSH and its precursors have been shown to decrease during the luteal phase (Draper et al., 2018), but no cortical changes in GSH measured by MRS have yet been reported, we extended the research to include the assessment of GSH.

## 2. Methods

### 2.1. Participants

Forty participants were initially recruited from 17 different sites. Sites collected data using GABA edited sequences that were readily available to them including HERMES (Chan et al., 2019), HERCULES (Oeltzschner et al., 2019), MEGA-PRESS (Mescher et al., 1998), and vendor specific sequences. Harmonisation across sequences was not possible at the time of data collection, and preprocessing of the data revealed issues with data quality and potential issues in combining across distinct scan protocols. Consequently, this analysis focuses on a subset of 30 participants collected across 12 sites using only HERMES or HERCULES approaches, which allow for the quantification of both GABA and GSH.

Participants included in this study met the following criteria: women aged 18–40 years, who had not used hormonal contraceptives for at least 3 months; were not currently or recently pregnant or breastfeeding; were free from diagnoses related to reproductive health such as endometriosis or polycystic ovarian syndrome; and who did not meet criteria for premenstrual dysphoric disorder (PMDD). Participants were all self-reported non-smokers. Additional screening was used to check if participants’ cycles were regular (defined as 28 +/− 5 days) and to confirm that participants experienced no symptom that could indicate a gynaecological health issue. Participants were assessed for PMDD using a subset of questions (questions 1:5 and A:E) from the Premenstrual Symptom Screening Tool (Steiner et al., 2003). No participants were found to meet the diagnostic criteria for premenstrual dysphoric disorder (PMDD), and only moderate symptoms were noted.

The average age of participants recruited for this study was 27 ± 5.2 years (range 18–39). The average cycle length was 28 days (range 24–34). Handedness as assessed by the Edinburgh Handedness Inventory (Oldfield, 1971) shows 27 participants were right handed, 1 ambidextrous and 2 left handed. All participants completed scans at three time points; however, the total number of scans included in the analysis varied slightly following data quality checks (details in Table 2).

### 2.2. Scheduling of scans

Each participant was scanned three times at three distinct points in their menstrual cycle.

In all but two instances, scans were collected in the following order from a consecutive cycle: early follicular; anticipated ovulation; and mid luteal phase. Early follicular scans were arranged to be 3 days post-menstrual bleeding; anticipated ovulation was calculated as average cycle length minus 14 days and, in all instances but one, this was confirmed with LH strips. Mid Luteal scans were scheduled as 22 days after the start of menstrual bleeding. The timings were selected to align with the following hormonal profiles: early follicular – low oestrogen, low progesterone; ovulation – approximate peak oestrogen, low progesterone; and mid luteal – peak progesterone, moderate oestrogen. Scans occurred 1 ± days around these windows to accommodate scheduling issues. If data collection from a consecutive cycle within a single cycle was not possible (e.g., due to scheduling issues or missed LH peak) scans were acquired from the missing time point within the next cycle. This occurred in 2/30 participants. Scans were collected at least one month after COVID-19 vaccination (when applicable).

### 2.3. Acquisition of MRS data

#### 2.3.1. Voxels

During each session MRS scans were acquired by placing a 3 * 3 * 3 cm sized voxel in the following locations: left motor cortex (LMC); and left insula (LINS); medial parietal cortex + midline dorsal posterior cingulate (MP); midline medial frontal + anterior midline cingulate cortex (MF). The order of acquisition of these voxels was fixed and as listed previously. For 1 participant data was only collected from the MF voxel. Each scan consisted of 320 transients (160 ON, 160 OFF), corresponding to approximately 10 minutes of scan time per region. The LMC voxel was centred over the hand knob region in left hemisphere and aligned with cortical surface in coronal plane to help avoid ventricle clipping. The LINS voxel was positioned in the left insula, centred on the circular sulcus, and it was aligned with the cortical surface/skull by rotating the voxel, and in the sagittal plane, the voxel is rotated to be parallel with the Sylvian fissure. The MP voxel was located in medial parietal cortex, and in the sagittal plane the voxel was rotated to align with connecting a line connecting the genu and splenium of the corpus callosum. The MF voxel was placed in the medial prefrontal cortex, and it was positioned medially on axial plane and superior to the genu of corpus callosum (aligning with corpus callosum).

#### 2.3.2. Scan parameters

Data were collected from 12 different sites, with each site collecting between 1 and 3 participants. As each participant acts as their own control and we are interested in changes occurring at the individual level, we did not fully harmonise scanning approaches across sites. The majority of data (11/12 sites, 27 participants) was acquired using a Hadamard encoding, and reconstruction of MEGA-edited spectroscopy (HERMES) sequence optimised for GABA and GSH (Chan et al., 2019). One site (3 participants) collected data using a Hadamard Editing Resolves Chemicals Using Linear-combination Estimation of Spectra (HERCULES) sequence (Oeltzschner et al., 2019). Both are optimised for quantifying GABA and GSH. For further details regarding scan parameters, please see Table S1.

### 2.4. Analysis of MRS data

All data were analysed using the Osprey software package (version 2.4.0) (Oeltzschner et al., 2020), following consensus-recommended pre-processing and linear-combination modelling procedures. For HERMES and HERCULES data, frequency- and phase-corrections were performed using probabilistic spectral alignment (Near et al., 2015). A Hankel singular value decomposition (HSVD) filter (Barkhuijsen et al., 1987) was subsequently applied to eliminate residual water signals and minimize baseline roll. The modelled spectral range was from 0.2 to 4.2 ppm. To establish the metabolite basis set, MRSCloud (Hui et al., 2022) was employed, encompassing a comprehensive collection of metabolites, including: ascorbate (Asc), aspartate (Asp), creatine (Cr), gamma-aminobutyric acid (GABA), glycerophosphocholine (GPC), glutathione (GSH), glutamine (Gln), glutamate (Glu), myo-inositol (mI), lactate (Lac), N-acetyl aspartate (NAA), N-acetyl aspartyl glutamate (NAAG), phosphocholine (PCh), phosphocreatine (PCr), phosphoryl ethanolamine (PE), scyllo-inositol (SL), and taurine (TAU). The sum spectrum included eight macromolecule (MM) basis functions (MM0.94, MM1.22, MM1.43, MM1.70, MM2.05, Lip0.9, Lip2.0). For co-edited MM peaks, specific designations were set at 1.2 and 1.4 ppm for GSH-edited difference spectra, and at 0.93 and 3.0 ppm for GABA-edited difference spectra. Amplitude-ratio soft constraints were applied to the amplitudes of the MM, lipids and NAAG/NAA peaks as defined in the LCModel manual (Provencher, 2021).

The default Osprey baseline knot spacing of 0.4 ppm was employed. Previous research (Zöllner et al., 2022) has demonstrated one-to-one amplitude-ratio soft constraint between the GABA amplitude and the co-edited MM at 3.0 ppm (referred to as ‘1–1 GABAsoft’ in Osprey) performs well. The water-reference data were modelled in the frequency domain with a six-parameter model (amplitude, zero- and first-order phase, Gaussian and Lorentzian line broadening, and frequency shifts).

Tissue differences in water relaxation (Wansapura et al., 1999) and MR visibility were addressed in quantification, using the (Gasparovic et al., 2006) approach implemented in Osprey. A further ‘alpha’ correction was applied to GABA estimates to account for established grey/white matter differences in GABA concentrations (Harris et al., 2015), and minimize the impact on results of scan-to-scan and subject-to-subject differences in voxel tissue composition; this procedure was not applied for GSH due to a lack of literature consensus GM/WM GSH differences and literature precedent for such a correction. Statistical analysis of voxel composition (segmented using SPM12) showed minimal systematic change in GM/WM proportions within voxels as a function of menstrual phase (Supplementary information).

### 2.5. Quality assurance (QA) of MRS data

#### 2.5.1. Data were checked using the following criteria

1. Visual Inspection: All spectra were visually inspected for the expected GABA and GSH peaks. Any spectra containing visible artifacts, such as out-of-voxel echoes (OOVs), were conservatively excluded.
2. Signal-to-Noise Ratio (SNR): The SNR was calculated to determine the strength of the signal compared to the background noise. Higher SNR values indicate better quality spectra. All included spectra met consensus-recommended (Wilson et al., 2019) data quality thresholds (i.e., <12 Hz linewidth).
3. Concentrations equal to 0 were interpreted as evidence of a failure to fit, and those data points were excluded from further analysis.

The number of data sets included out of the total collected, can be seen in Table 2.

### 2.6. Data analysis

Multilevel modelling was used to analyse the data, as this allowed for all data sets passing QA to be maintained and for potential site variability to be factored in. Three-level (i.e., menstrual phase nested within participants nested within sites) random-effects ANOVA models were estimated for GABA and GSH measurements from each of the four evaluated brain regions (i.e., 8 models total) using the Linear Mixed Models module of IBM SPSS Statistics v27 software (IBM, Armonk NY), which includes all available data via Restricted Maximum Likelihood (REML) estimation (Heck et al., 2022). First, unconditional means models, with no predictors and scaled identity covariance matrices specified at each level, were estimated to decompose variance in GABA and GSH measurements across levels, providing intraclass correlation coefficients (ICC) at each level of the data hierarchy. Next, the fixed effect of menstrual cycle phase (i.e., early follicular vs. anticipated ovulation vs. mid luteal phase) was entered into the above models, with the covariance matrix at level 1 changed from scaled identity to diagonal (i.e., the default) to improve model estimation by allowing unequal variances across menstrual phase. To control for alpha inflation due to multiple testing, we held each of the 8 evaluated tests to a nominal alpha level of 0.01. Although not conducted a priori, we estimated the minimum detectable effect size with ≥ 80 % power by region using the sensitivity analysis function of G*Power 3.1 (Faul, Erdfelder, Lang, et al., 2007).

## 3. Results

Linear Mixed Models. Results from unconditional means models are provided in Table 3. For GABA, with the exception of MP for which ICC values were roughly equivalent across levels, most of the observed variance was distributed relatively equally between levels 1 (menstrual phase) and 3 (sites). For GSH, although the model for MF converged, it produced a Final Hessian Matrix that was not positive definite, with the ICC for level 2 fixed to “0” to enable model estimation; estimated ICCs for levels 1 and 3 were roughly equivalent. Remaining GSH unconditional means models attributed most of the total variance to levels 1 and 3, broadly similar to what was found for GABA. In sum, with the notable exception of MP GABA, most GABA and GSH variance was roughly equally divided between levels 1 (menstrual phase) and 3 (sites), with relatively little variance attributed to level 2 (participants).

**Fig. 1.**
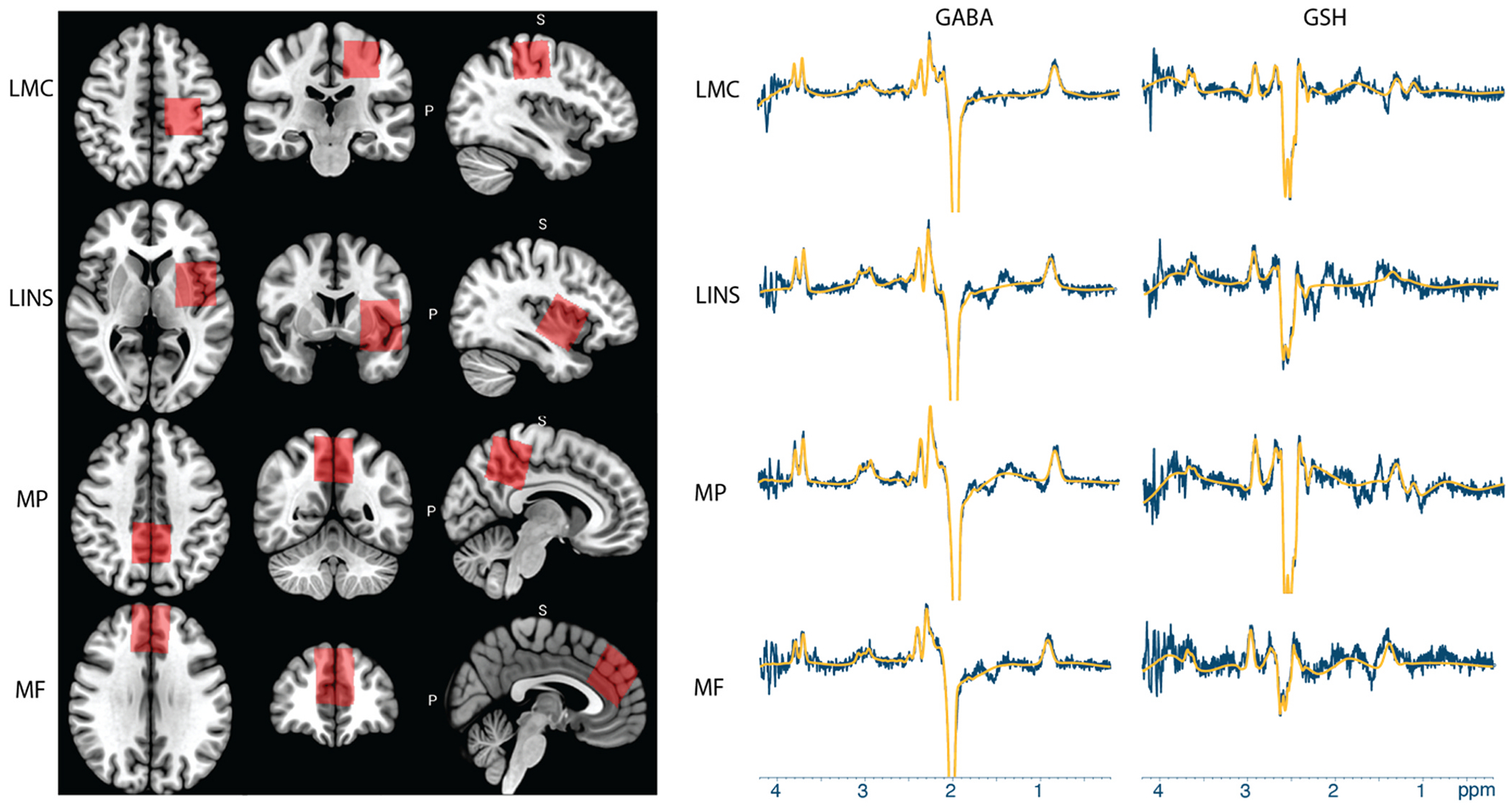
Axial, coronal and sagittal sections of a representative participant showing voxel positioning for midline medial frontal + anterior midline cingulate cortex (MF), medial parietal cortex + midline dorsal posterior cingulate (MP), left motor (LM) and left insula (LINS) locations. Example spectral fits for each metabolite at voxel for GABA and GSH presented on the right.

Results from random effects ANOVA models, with the fixed effect of menstrual cycle phase estimated separately for each combination of metabolite and brain region, are provided in Tables 4 and 5. Effects of menstrual cycle phase on GABA (*p* = 0.19 to *p* = 0.971) and GSH (*p* = 0.37 to *p* = 8.8) levels were uniformly non-significant, providing no evidence for non-zero associations of menstrual cycle phase with brain GABA or GSH levels. The distribution of data for GABA and GSH can be seen in Fig. 1.

Results from sensitivity analyses are presented in Table S3. These analyses determined that, for most models (i.e., GABA: LMC, MF, LINS; GSH: LMC, MP), we had sufficient power to detect effect sizes of small-medium or larger magnitude. Remaining models (i.e., GABA: MP; GSH: LINS, MF) had sufficient power to detect effect sizes of medium or larger magnitude.

## 4. Discussion

Our findings suggest that MRS measures of GABA and GSH do not systematically vary as a function of the menstrual cycle phase in healthy women. These null effects are consistent across all four cortical regions studied, suggesting a lack of widespread change in cortical levels of GABA or GSH. These findings contrast with previous work in smaller sample sizes (N = 7), which found higher GABA in the follicular phase of the cycle compared to the luteal phase (De Bondt et al., 2015; Epperson et al., 2002, 2005), and higher GABA around ovulation (Harada et al., 2011). Instead, they are more aligned with work by Chrzan et al. (2013) in which no systematic changes in GABA were identified.

There are several ways in which we aimed to build on previous MRS studies of potential menstrual cycle effects. In our participant inclusion, we were cautious to exclude women who smoked or had any nicotine intake, as Epperson et al. (2005) reported no cyclic changes in their sample of women who smoked (N = 4–5). We were also cautious to exclude women meeting criteria for PMDD, as another study by the same group (Epperson et al., 2002) suggested those with PMDD may show a reversal in GABA-related changes. We also sought to improve our MRS measures by acquiring more transients than previous work to yield higher SNR. Additionally, we sought to expand the sample size used in previous work, reaching an N of 26–29 depending on voxel. The previous studies did not report effect sizes; however, calculations of these based on available means/SDs reveal very large effect sizes (Hedges G values of.97 to 2.1), which should be detectable at moderate sample sizes such as the ones used in this study. Failure to detect these large effects despite our increased sample size may highlight issues relating to the small samples included in the original study and consequently inflated effect sizes (e.g. see Button et al. 2013). If this is the case, previous findings of changes in MRS measures of GABA may be either incorrect or overstated.

Exactly which aspects of GABA are being measured by MRS is still a little unclear. A lack of correlation between MRS measures of GABA and measures of GABA-A synaptic activity with transcranial magnetic stimulation (TMS) had previously led to conclusions that MRS measures of GABA primarily relate to extra-synaptic tone (Dyke et al., 2017; Stagg et al., 2011; Stagg, Bestmann, et al., 2011). However, more recent work has identified an association between the two measures (A. D. Harris et al., 2021), and speculatively, it seems most likely that for GABA, glutamate and other neuro-metabolites of interest such as GSH, what we are measuring reflects contributions across many cellular compartments in addition to extracellular pools (see Mullins 2018 for discussion). This consideration is raised to highlight why/how differences in findings may arise when considering menstrual cycle-related changes quantified using different measurement approaches. For example, in a TMS study of menstrual cycle effects in a sample of 13 women, GABAergic inhibition was higher in the luteal phase compared to the follicular phase (Smith et al., 1999), which conflicts directly with previous MRS work (De Bondt et al., 2015; Epperson et al., 2002, 2005) in which MRS measures of GABA were higher during the follicular phase. Further discrepancies exist between findings from MRS measures of GABA change (Epperson et al., 2002) and older work exploring changes in plasma measures of GABA in 21 women without PMDD (Halbreich et al., 1996). It is important to consider that although GABA has been seen to fluctuate with the menstrual cycle in other modalities, MRS measures of GABA may be more robust to these changes as a consequence of differences in specificity.

## 5. Limitations and considerations

We accept the possibility that the addition of site-related variability within our dataset may reduce our power to detect effects. However, it should be noted that systematic variability attributed by site (for example, as a consequence of subtle differences in scanning protocols) should be partially controlled, as the same participants were tested three times, and our interest is in quantifying that within-subject change. Additionally, effect sizes from previous work were large and consequently should require less power to identify.

We were also unable to quantify oestradiol and progesterone levels in our approach, so cannot verify that timing corresponded exactly to peaks and troughs in these hormones. However, all but one participant had their approximate ovulation and luteal phase scans scheduled in accordance with a positive peak in LH, which should give confidence in the timing.

Although our sample size is larger than previous work, we acknowledge that it is still modest. We note that our analysis suggests we had sufficient power to observe ≥ small-medium effects for GABA in LMC, MF and LINS voxels and ≥ medium effects in MF. For GSH we had power to detect ≥ small-medium effects for LMC and MP and ≥ medium effects in LINS and MF. It therefore remains a possibility that we failed to detect smaller effects, particularly in our MF voxel. However, we argue that if such small effects are present, they are unlikely to influence the majority of MRS research in any meaningful way.

## 6. Conclusions and recommendations

Our data do not support previous reports of systematic change in GABA as a consequence of the menstrual cycle. Nor do they show any systematic variability in GSH. Coupled with the knowledge that participants in MRS studies tend to be young adults (a demographic shown in some geographical regions to use hormonal contraceptives more frequently (Firman et al., 2018; M.L. Harris et al., 2020)) drawn from university populations, we do not recommend the exclusion of women in MRS studies and suggest any variability attributed to MRS measures of GABA or GSH as a consequence of this is likely to be negligible.

## Supplementary Material

Supplementary materials

## Figures and Tables

**Fig. 2.**
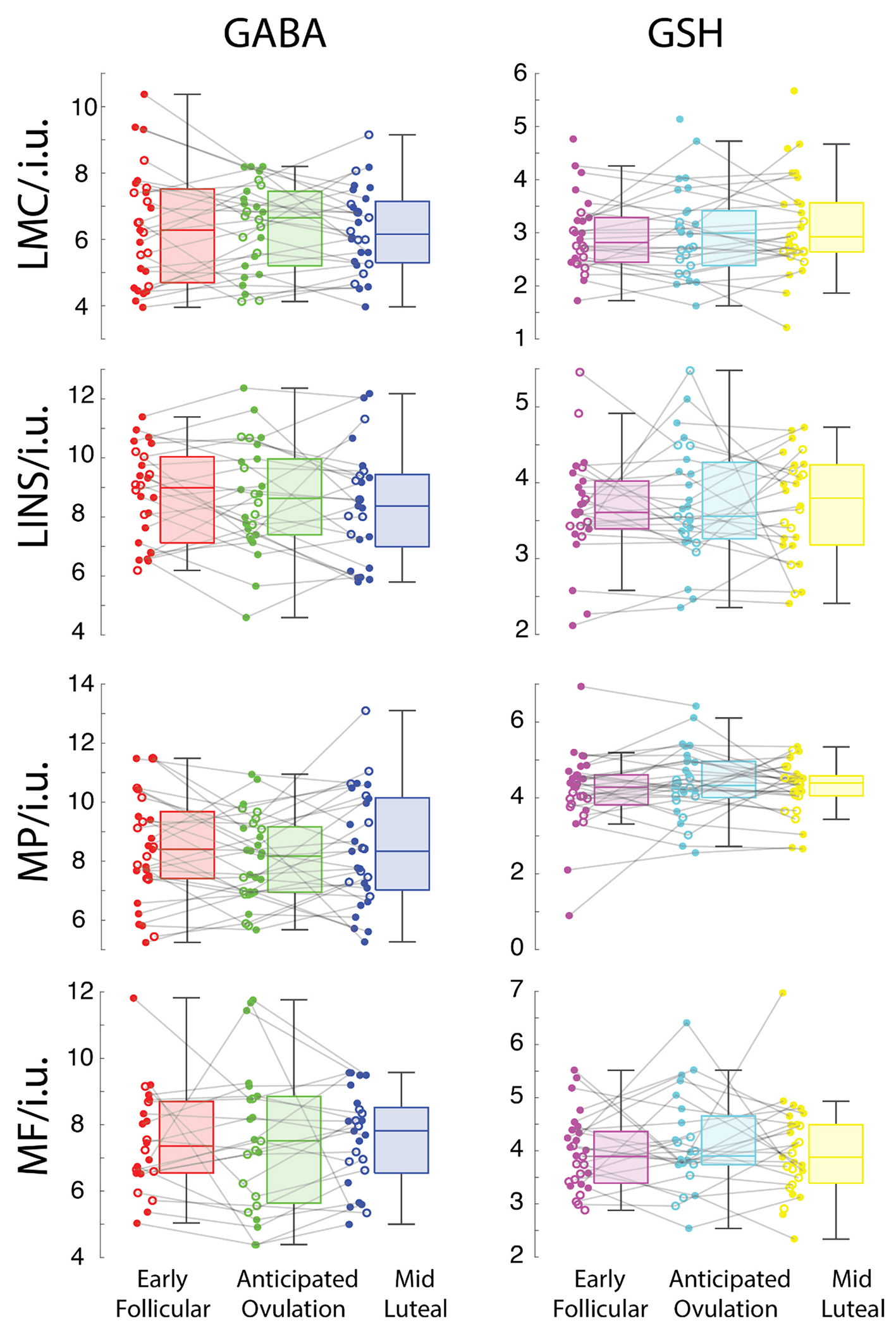
Distribution plots of metabolite levels in four brain regions (LMC, LINS, MP, and MF) across three time points (Early Follicular, Anticipated Ovulation, Mid Luteal). The left panel shows GABA data, and the right panel shows GSH data. Each panel includes individual data points and boxplots, where the central mark represents the median, the edges of the box represent the 25th and 75th percentiles, and the whiskers extend to the most extreme data points. Grey lines connect data points from the three time points for each subject. All visualizations are color-coded: GABA data (red, green, blue) and GSH data (pink, cyan, yellow). Filled data points represent Philips data, while open circles indicate Siemens data.

**Table 1:** Summary of studies assessing MRS-GABA across different phases of the menstrual cycle (within-subjects).

Reference	Sample	Measured region	Scan Info	Results
De Bondt et al., 2015	N = 11 Age: 24.37 ± 3.6	Prefrontal cortex in dominant hemisphere Voxel size: 2*2*2 cm	3T; Unspecified edited sequence. TR: 1.5s TE: 70 ms Averages: 100	Increased GABA+ around LH peak (periovulatory period) compared to luteal and early follicular phases. Higher but more variable oestradiol during ovulation than follicular. Elevated but less variable in luteal. No correlations between GABA+ measures and any hormone measures.
Harada et al., 2011	N = 7 Age: unclear	L frontal lobe Lentiform nucleus Anterior cingulate Voxel size: 3*3*3 cm	3T; MEGA-PRESS TR: 1.5s TE: 68ms Averages: unclear	Increased GABA+ during mid follicular phase compared to mid luteal in frontal lobe and lentiform nucleus but not anterior cingulate.
Epperson et al., 2002	N = 14 Age: 30.1 (21-45) N = 9 PMDD Age: 34.6 (26-39)	Occipital cortex Voxel size: 1.5*3*3 cm	2.1T; J-Editing TR: 3.39s TE : 68 ms Averages: unclear	Increased GABA+ during mid follicular phase in comparison to mid and late luteal in women without PMD. Increased GABA+ during late luteal compared to mid follicular in women with PMDD.
Epperson et al., 2005	N = 11 Age: 30.8 ± 5.9 N = 6 smokers Age: 35 ± 13	Occipital cortex Voxel size: 1.5*3*3 cm	2.1T; J-Editing TR: 3.39s TE: 68 ms Averages: unclear	Increased GABA+ during follicular phase in comparison to mid luteal in non-smokers. No change in smokers.
Chrzan et al., 2013	N = 11 Age: 24.3 ± 3.8	R & L frontal lobe R & L basal ganglia R & L occipital lobe Voxel size: 2*2*2cm	1.5 T; PROBE-P TR: 1.5s TE: 35ms Averages: unclear	No significant changes in GABA+ between early follicular, approximate ovulation and late luteal phases. Regional differences found for Lac, NAA and Glx across different time points.

PMDD = premenstrual dysphoric disorder; Lac = lactate; NAA = N-acetylaspartate ; GABA+ =Gamma-aminobutyric acid + macromolecules; GlX = Glutamine + glutamate; L = Left; R = Right. Age reported as mean and standard deviation whenever possible. Scanner information includes field strength and acquisition protocol when available. Note: sample size reflects number included in statistical analysis.

**Table 2:** Participant demographics for each voxel location

	N	N full data sets	N Included Scans	Age	Cycle length
**LMC (GABA)**	28	26	81 (of 84)	26.9 ± 5.3	28.8 ± 2.5
**Lins (GABA)**	28	21	75 (of 84)	26.9 ± 5.3	28.9 ± 2.4
**MP (GABA)**	29	29	87 (of 87)	26.9 ± 5.2	28.7 ± 2.4
**MF (GABA)**	26	18	69 (of 78)	26.7 ± 5.2	28.7± 2.5
**LMC (GSH)**	28	25	79 (of 84)	26.9 ± 5.3	28.8 ± 2.5
**Lins (GSH)**	28	22	78 (of 84)	26.9 ± 5.3	28.9 ± 2.4
**MP (GSH)**	29	29	87 (of 87)	26.9 ± 5.2	28.7 ± 2.4
**MF (GSH)**	29	23	79 (of 87)	26.4 ± 5.0	28.8 ± 2.5

N = participants included in analysis. N full data sets = participants with all 3 scans. N included scans = total scans entered into analysis from possible total calculated as N*3. Age and cycle length reported as mean and SD

**Table 3:** ICC values from unconditioned means models.

	*Level 1: Menstrual Phase*	*Level 2: Participant*	*Level 3: Site*
**GSH**			
LMC	0.151	0.197	0.653
LINS	0.475	0.144	0.381
MP	0.350	0.103	0.547
MF	0.480	0	0.520
**GABA**			
LMC	0.353	0.053	0.594
LINS	0.343	0.185	0.472
MP	0.303	0.375	0.322
MF	0.490	0.024	0.486

* Note: Model converged, with a Final Hessian Matrix that was not positive definite. ICC for level 2 fixed to “0” to enable model estimation.

**Table 4:** Random effects ANOVA models for GABA with the fixed effect of menstrual cycle phase.

Voxel		*Num df*	*Denom df*	F	P
LMC	Intercept	1	9.963	296.072	< 0.001
	Phase	2	36.757	0.104	0.902
LINS	Intercept	1	10.771	470.919	< 0.001
	Phase	2	26.375	0.928	0.408
MP	Intercept	1	10.629	513.529	< 0.001
	Phase	2	28.409	1.773	0.188
MF	Intercept	1	12.411	440.953	< 0.001
	Phase	2	33.773	0.029	0.971

**Table 5:** Random effects ANOVA models for GSH with the fixed effect of menstrual cycle phase.

Voxel	Num df	*Num df*	*Denom df*	F	P
LMC	Intercept	1	9.812	188.743	< 0.001
	Phase	2	28.704	1.031	0.369
LINS	Intercept	1	10.297	508.302	< 0.001
	Phase	2	30.238	0.208	0.814
MP	Intercept	1	10.478	447.639	< 0.001
	Phase	2	38.116	0.862	0.431
MF	Intercept	1	10.497	381.981	< 0.001
	Phase	2	39.494	0.131	0.878

## Data Availability

Data will be made available on request.

## Appendix A. Supporting information

Supplementary data associated with this article can be found in the online version at doi:10.1016/j.jneumeth.2025.110430.

## Abbreviations:

GABA: Gamma-aminobutyric acid
GSH: glutathione
MRS: magnetic resonance spectroscopy
HERMES: Hadamard Encoding and Reconstruction of MEGA-Edited Spectroscopy, menstrual cycle
