# Supplementary material for: Magnetic resonance spectroscopy and the menstrual cycle: A multi-centre assessment of menstrual cycle effects on GABA & GSH
Source: J Neurosci Methods. Author manuscript; Available in PMC 2026 Jul 7. (PMC13339251; doi:10.1016/j.jneumeth.2025.110430)
Supplement: Supplementary materials [file NIHMS2188030-supplement-Supplementary_materials.docx]

**Table S.1.** Scan parameters for each site included in the analysis. Including number of participants collected (N), and number of brain regions scanned per participant (vox).

| **Site name/ID Participants/ Voxels** | **3T Scanner vendor/ model** | **Tx/Rx hardware** | **B0 shimming approach** | **Sequence variant** | **Phase cycling** | **TE (ms)** | **TR (ms)** | **Assumed water ref frequency**  **(ppm)** | **Editing pulse duration (ms)** | **FWHM bandwidth**  **(spect bandwidth Hz)** | **Water suppression** | **Data points** |
| --- | --- | --- | --- | --- | --- | --- | --- | --- | --- | --- | --- | --- |
| Bangor University  **BU (1)** N=3; Vox=4 | Phillips Elition X | Tx- Body Coil, 32 channel Head coil | PB Volume  (Order: second) | HERCULES  JHU patch | 16 | 80 | 2000 | 127.726 | 20 | Missing info | VAPOUR | 2048 |
| Ewha W. University / **EBI (2)**  N=3; Vox=4 | Philips Ingenia CX | 32 channel head coil | PB-auto (Order: second) | HERMES JHU patch | 16 | 80 | 2000 | 4.68 | 20 | 2000 | MOIST (window = 140 Hz) | 2048 |
| John Hopkins  **JHU (3)**  N= 1; Vox =4 | Philips Ingenia Elition X scanner | quadrature transmit body coil and 32-channel head coil | PB-auto (Order: first) | HERMES  JHU patch | 16 | 80 | 2000 | 4.68 | 20 | 2000 | CHESS | 2048 |
| Katholieke Universiteit Leuven **KUL (4)**  N= 3;Vox-=4 | Philips / Achieva dStream | 32channel head coil | PB-auto (Order: second) | HERMES JHU patch | 16 | 80 | 2000 | 4.68 | 20 | 2000 | MOIST (window = 140 Hz) | 1024 samples |
| University of Florida  **UFl (5)**  N= 2;Vox= 4 | Philips MR7700 | quadrature transmit body coil and 32-channel phased array receive head coil | PB-auto (Order: first) | HERMES JHU patc | 16 | 80 | 2000 | 4.68 | 20 | 2000 | CHESS | 2048 |
| Hasselt University, **UHa (6)**  N= 3; Vox = 4 | Philips / Achieva dStream | 32 channel head coil | PB-auto (Order: second) | HERMES JHU patch | 16 | 80 | 2000 | 4.68 | 20 | 2000 | MOIST (window = 140 Hz) | 1024 samples |
| University of Nottingham  **UoN (7)**  N= 3; Vox = 4 | Philips Ingenia | 32 channel head coil | PB-auto (Order: not specifiable) | HERMES JHU patch | 16 | 82 | 2000 | 4.68 | 20 | 2000 | CHESS | 2048 |
| University of Cambridge  **CU (8)**  N= 3; Vox = 4 | Siemens Magnetom Prisma fit | 32 channel head coil | Brain (GRE) | mgs_svs_ed HERMES | Auto | 80 | 2000 | 4.68 | 20 | 2000 | Water saturation (BW 50Hz) | 2048 |
| Perdue  **PU (9)**  N= 3; Vox= 4 | Siemens MAGNETOM Prisma | 64 channel head coil | Brain (GRE) | HERMES JHU | Auto | 80 | 2000 | 4.68 | 20 | 2000 | Water saturation (BW 50Hz) | 2048 |
| University of Coimbra  **UC (10)**  N= 3; Vox= 4 | MAGNETOM Prisma Fit, syngo XA30 | 64 channel head coil | Advanced | HERMES JHU patch | Auto | 80 | 2000 | 4.68 | 20 | 2000 | Water saturation (BW 50Hz) | 2048 |
| University of Oregon  **UO (11)**  N= 2; Vox= 4 | Siemens, Prisma | 32 channel head coil | Brain (GRE) | HERMES | Auto | 80 | 2000 | 4.68 | 20 | 2000 | Water saturation BW 50 Hz | 2048 |
| Keio University  **KOU (12)**  N=1; Vox =1 (MF) | Siemens MAGNETOM Prisma | 32ch head coil | Missing info | HERMES | Auto | 80 | 2000 | Missing info | Missing info | Missing info | Missing info | 2048 |

**Grey/white matter fraction as a function of menstrual cycle**

Several analyses were conducted to explore the potential impact of the menstrual cycle on voxel composition. Although GABA values were corrected for tissue fraction (see Methods. Analysis of MRS data for details), this was not possible for GSH making establishing that there were no systematic changes an important issue to explore. Grey and white matter fractions across the three different phases of the menstrual cycle are plotted for each voxel in Figure S.1.


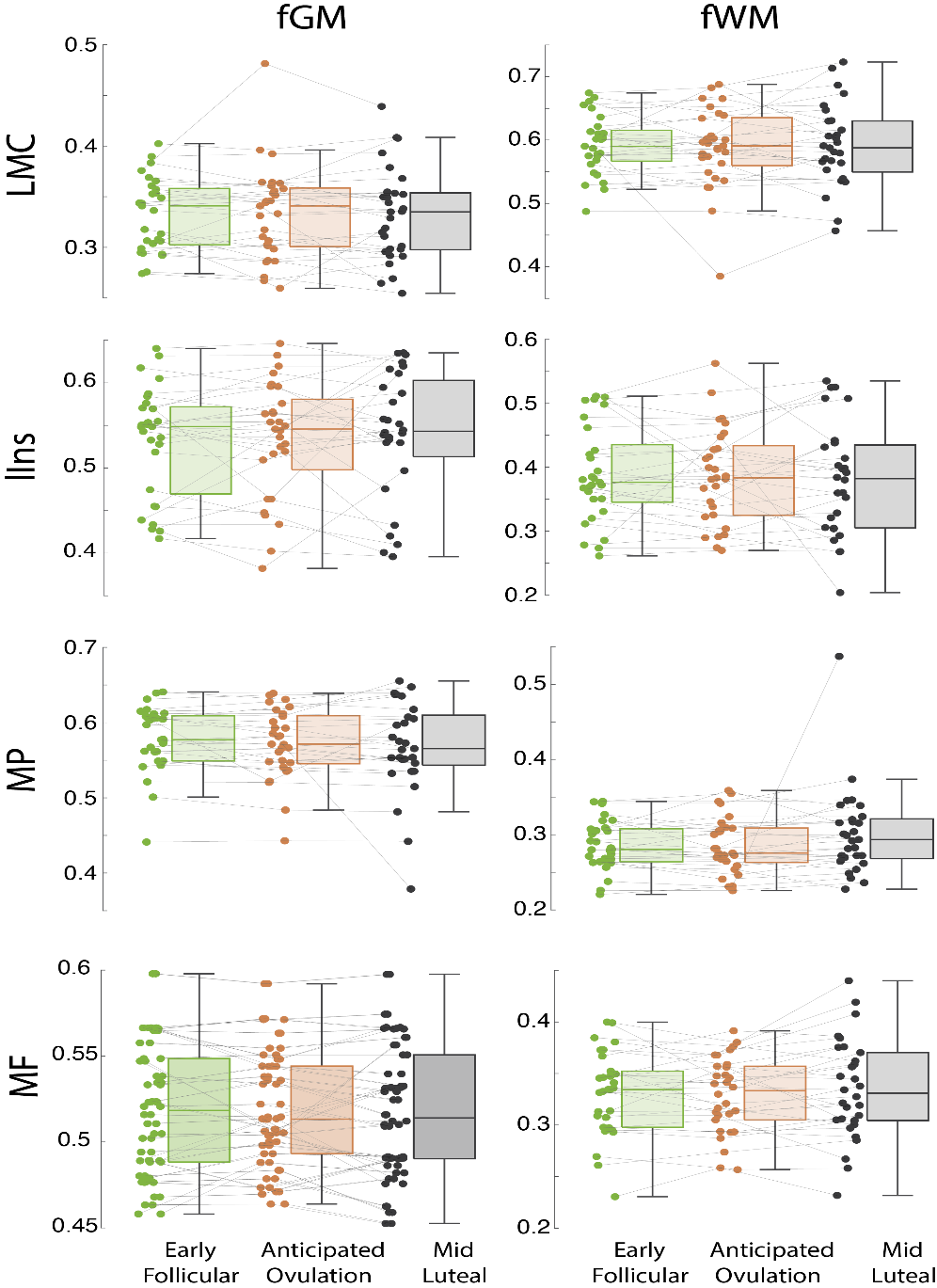


**Figure S.1:** Distribution plots of white and grey matter proportions within four voxels (LMC, LINS, MP, and MF) across three time points (Early Follicular, Anticipated Ovulation, Mid Luteal). The left panel shows fraction of grey matter, and the right panel shows fraction of white matter. Each panel includes individual data points and boxplots, where the central mark represents the median, the edges of the box represent the 25^th^ and 75^th^ percentiles, and the whiskers extend to the most extreme data points. Grey lines connect data points from the three time points for each subject.

**Statistical analysis of voxel composition:** Repeated measures ANCOVAs were run for each voxel placement (LMC, LINS, MP, MF) and tissue type (grey matter -GM, white matter -WM) with site entered as a covariate to assess if there were any systematic changes in tissue composition across scans acquired during the three different phases of the menstrual cycle. Greenhouse Geisser correction was applied in instances where Munchleys test revealed violation of the assumption of sphericity. All analysis in this section were run using SPSS Statistics V28 software (IBM, Armonk NY).

No significant changes in grey matter fraction were identified in any voxel. For white matter fractions no significant differences were found for the LINS, MF and MP voxels (Table S.2). However, a significant effect of time (menstrual cycle phase) was identified for the LMC voxel (f(2,24)=3.36, p=.042,



ηp2 = .111), in addition to a significant interaction between the repeated measures variable of time and the covariate of site (f(2,24)=3.44,p=.039. ηp2= .113). The low number of cases (1-3) per site poses challenges to further statistical exploration of this effect, however, this effect largely appears to be driven by 2 participants from one site which show much higher variability in white matter quantity between scans than the norm (Figure S.2). Notably, removal of either of these participants from the analysis results in no significant differences being identified.

**Table S.2.** Results of ANOCOVAs with scan time entered as a within subjects’ factor and site as a covariate.

|  | **DF** | **F** | **P** | **ηp2** |
| --- | --- | --- | --- | --- |
| **Grey matter** |  |  |  |  |
| LMC (time) | 1.967,53.101 | 2.748 | .073 | .092 |
| LMC (time*site) | 1.967,53.101 | 3.083 | .055 | .102 |
| LINS (time) | 2,52 | .034 | .967 | .001 |
| LINS (time*site) | 2,52 | .208 | .813 | .008 |
| MP (time) | 2,54 | .439 | .647 | .016 |
| MP (time*site) | 2,54 | .310 | .734 | .011 |
| MF (time) | 2,56 | .338 | .715 | .012 |
| MF (time*site) | 2,56 | .498 | .611 | .017 |
| **White matter** |  |  |  |  |
| LMC (time) | 2,54 | 3.360 | .042* | .111 |
| LMC (time*site) | 2,54 | 3.442 | .039* | .113 |
| LINS (time) | 2,52 | .206 | .815 | .008 |
| LINS (time*site) | 2,52 | .293 | .747 | .011 |
| MP (time) | 1,366,36.941 | 2.127 | .147 | .073 |
| MP (time*site) | 1,366,36.941 | .694 | .453 | .025 |
| MF (time) | 2,56 | .893 | .415 | .031 |
| MF (time*site) | 2,56 | 1.292 | .283 | .044 |

**
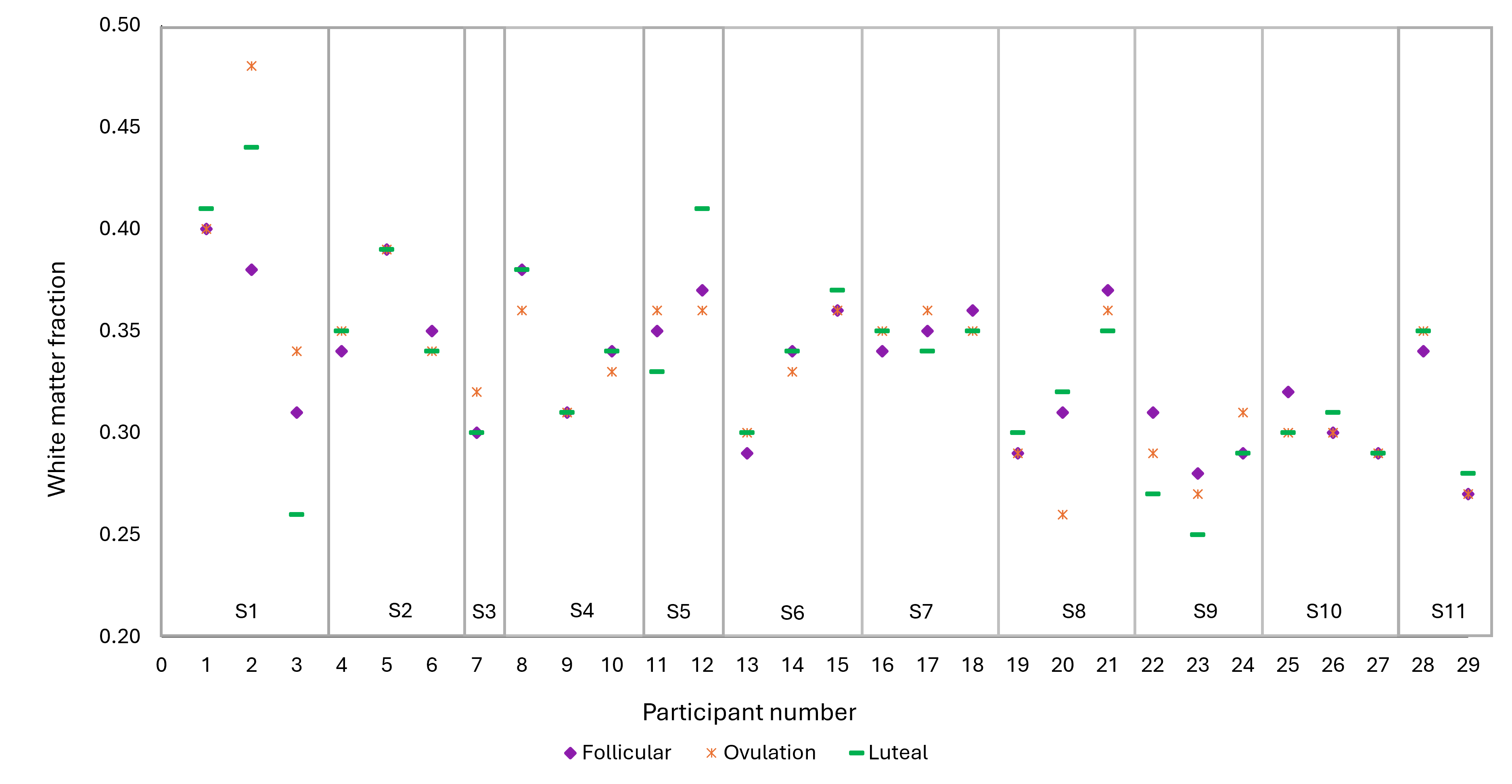
**

**Figure S.2.** White matter fractions for LMC voxel across time points. Data from follicular scans in purple, ovulation in orange and luteal in green. Individual participant numbers displayed on x axis with additional notation indicating site number (e.g. S1 representing site 1, S2 as site 2 etc). Grey lines divide individual participants into groups by site.

**Accuracy of voxel placement:** In the absence of robust evidence supporting systematic change in tissue fraction as a function of the menstrual cycle (above) we ran partial correlations (controlling for site) as a proxy measure of accuracy of voxel placement across the three scans. These correlations were highlight significant across all voxels and time points for both grey and white matter. Grey matter fractions within the LMC voxel were highly correlated across the three (early follicular, approximate ovulation and luteal) time points (min r= .767, max r= .856, all p <.001). The same was true for LINS voxel (min r= .688, max r= 817, all p<.001); the MP voxel (min r= .888, max r= .908, all p<.001); and the MF voxel (min r= .872, max r = 919, all p<.001). White matter fractions were also significantly correlated across scans for the LMC voxel (min r=.625, max r= 837, all p<.001); the LINS voxel (min r= 645, max r= 790, all p <.001); the MP voxel (min r= .623, max r= .897, all p<.001); and for the MF voxel (min r= .857, max r= .893, all p<.001). Individual correlations visible in Table.S.3.

In the absence of robust systematic changes in voxel composition over the menstrual cycle, we interpret these large correlations as indicators of voxels being placed consistently across the three testing sessions.

|  | **df** | **R** | **P** |
| --- | --- | --- | --- |
| **Grey matter** | | | |
| LMC 1 and 2 | 26 | .767 | <.001 |
| LMC 2 and 3 | 26 | .811 | <.001 |
| LMC 1 and 3 | 26 | .856 | <.001 |
| LINS1 and 2 | 25 | .817 | <.001 |
| LINS 2 and 3 | 25 | .688 | <.001 |
| LINS 1 and 3 | 25 | .780 | <.001 |
| MP 1 and 2 | 26 | .888 | <.001 |
| MP 2 and 3 | 26 | .889 | <.001 |
| MP 1 and 3 | 26 | .908 | <.001 |
| MF 1 and 2 | 27 | .919 | <.001 |
| MF 2 and 3 | 27 | .872 | <.001 |
| MF 1 and 3 | 27 | .907 | <.001 |
| **White matter** | | | |
| LMC 1 and 2 | 26 | .625 | <.001 |
| LMC 2 and 3 | 26 | .790 | <.001 |
| LMC 1 and 3 | 26 | .837 | <.001 |
| LINS1 and 2 | 25 | .790 | <.001 |
| LINS 2 and 3 | 25 | .645 | <.001 |
| LINS 1 and 3 | 25 | .782 | <.001 |
| MP 1 and 2 | 26 | .897 | <.001 |
| MP 2 and 3 | 26 | .633 | <.001 |
| MP 1 and 3 | 26 | .623 | <.001 |
| MF 1 and 2 | 30 | .893 | <.001 |
| MF 2 and 3 | 30 | .857 | <.001 |
| MF 1 and 3 | 30 | .870 | <.001 |

**Table S.3.** Partial correlations (controlled for site) for each voxel across each time point.

**Post hoc sensitivity analysis:** Using our given sample size and mean correlation among repeated measures we estimated the minimum detectable effect size with ≥80% power by region using the sensitivity analysis function of G*Power 3.1 (Faul, Erdfelder, Lang, et al., 2007). Our results show that in most regions we had power to detect "small to medium" effect sizes at minimum for GABA (the exception being MF, in which we had power to detect "medium" effect sizes). For GSH, we had power to detect "small to medium" effect sizes in LMC and MP and "medium" effect sizes in LINS and MF (Table S.4).

**Table S.4. Results of sensitivity analysis**

|  | GABA r | req f | GSH r | req f |
| --- | --- | --- | --- | --- |
| LMC | 0.681 | 0.197 | 0.762 | 0.170 |
| LINS | 0.717 | 0.185 | 0.489 | 0.249 |
| MP | 0.710 | 0.184 | 0.606 | 0.215 |
| MF | 0.470 | 0.264 | 0.413 | 0.262 |
